# Analysis of Disaster-Related Deaths in the Great East Japan Earthquake: A Retrospective Observational Study Using Data from Ishinomaki City, Miyagi, Japan

**DOI:** 10.3390/ijerph19074087

**Published:** 2022-03-30

**Authors:** Motohiro Tsuboi, Manabu Hibiya, Rumiko Tsuboi, Shigemasa Taguchi, Koichi Yasaka, Kazuya Kiyota, Kayako Sakisaka

**Affiliations:** 1 Graduate School of Public Health, Teikyo University, 2-11-1 Kaga, Itabashi-ku, Tokyo 173-8605, Japan; hibiya.manabu.uy@teikyo-u.ac.jp (M.H.); sakisaka@med.teikyo-u.ac.jp (K.S.); 2 Department of Emergency and Critical Care Medicine, Japanese Red Cross Saitama Hospital, 1-5 Shintoshin, Chuoh-ku, Saitama-City 330-8553, Japan; tagutin1977@gmail.com (S.T.); yasaka51@gmail.com (K.Y.); kiyota_ind@mac.com (K.K.); 3 Department of Teikyo Academic Research Center, Teikyo University, 2-11-1 Kaga, Itabashi-ku, Tokyo 173-8605, Japan; 4 Department of Gastroenterology, Jichi Medical University Saitama Medical Center, 1-847 Amanumacho, Omiya-ku, Saitama-City 330-0834, Japan; kobayashi.rumiko@gmail.com; 5 Faculty of International Liberal Arts, Kaichi International University, 1225-6 Kashiwa, Kashiwa-City 277-0005, Japan

**Keywords:** disaster, disaster-related health, nursing care, indirect health effect, Great East Japan Earthquake

## Abstract

Disaster-related deaths are of two types: direct and indirect. Preventable disaster-related deaths reported in the Great East Japan Earthquake (GEJE) included a large number of indirect deaths. This study aimed to investigate the data on disaster-related deaths in the GEJE in Ishinomaki City, Miyagi Prefecture, and to clarify the scope of disaster-related deaths to help future disaster preparedness. A retrospective observational study was conducted using public data on disaster-related deaths from March 2011 to January 2021, available at Ishinomaki City Hall. Descriptive and Cox regression analyses were conducted. The most common direct cause of disaster-related deaths was respiratory diseases, which were more common among those aged less than three months and over 60 years. Suicide was common among those aged under 60 years, and the proportion increased more than six months after the disaster. The risk of death was significantly higher among those who needed nursing care than among those independent in daily living. The results indicate that measures should be taken for the elderly and those who need care from an early phase after the disaster. The analysis of data on disaster-related deaths in other affected municipalities may provide further evidence to help reduce disaster-related deaths.

## 1. Introduction

Disaster deaths include “direct” and “indirect” deaths [1]. “Direct” deaths are caused by physical events such as earthquakes, tsunamis, and radiation exposure; meanwhile, “indirect” deaths are caused by secondary health issues resulting from disasters and relate to inadequate medical systems and poor evacuations [2,3]. Notably, indirect deaths have been found to occur not only during the acute phase of a disaster but also over the long term [4]. In addition, the indirect health effects of disasters mainly appear among people who are socially vulnerable [5]. For example, a review of the health effects of hurricane disasters in the USA report long-term and significant indirect effects among vulnerable populations, such as the bedridden elderly, patients with chronic diseases, and homeless and racialized populations with low socioeconomic status (SES) [6]. However, although disaster medical responses based on objective data and information are necessary to reduce deaths associated with these indirect effects, medical data for large-scale natural disasters are insufficient [7,8,9]. Furthermore, a systematic review advised that improving the response capacity of medical support teams and post-disaster primary health care in terms of preparedness and actual medical intervention is essential to reduce the risk of indirect deaths during disasters; however, this initiative requires quantitative data collection and analysis [10,11].

To date, most reports of indirect deaths in large-scale natural disasters worldwide have been described at the anecdotal level [12]. However, in Japan, indirect deaths caused by disasters have been called disaster-related deaths since the Great Hanshin-Awaji Earthquake of 17 January 1995 [13,14]. After the Great East Japan Earthquake (GEJE) of March 2011, a large number of disaster-related deaths were announced by the Japanese government [15]. As of March 2021, 3774 disaster-related deaths were reported to have been caused by GEJE [16]. In GEJE, 14.0% of the deaths within three weeks of the disaster were preventable disaster deaths, most of which were indirect deaths caused by problems in the medical delivery system, such as lack of medical supplies and delays in medical intervention, and poor evacuation environments [5,17]. While it is very important to determine how to reduce such indirect deaths during disasters, no detailed analyses using data on disaster-related deaths in Japan have yet been conducted.

Ishinomaki City, Miyagi Prefecture, located on the Pacific coast near the epicenter of the earthquake, was one of the hardest affected areas in the GEJE and was the site of a multiple disaster caused by an earthquake and tsunami. Specifically, 13.2% of Ishinomaki City was flooded by the tsunami, and 76.6% of houses were damaged [18]. As of October 2021, 3277 direct deaths and 276 disaster-related deaths have been reported in Ishinomaki City [18]. This study analyzed the disaster-related deaths in Ishinomaki City to clarify the status of indirect deaths during such a large-scale disaster.

The authors are health professionals who have been involved in support and research activities in disaster medicine in Japan. Based on our experience and knowledge of the disaster area, we believe that research on disaster-related deaths from the GEJE will be useful in clarifying the characteristics of indirect deaths and, ultimately, in reducing preventable disaster-related deaths in the future. Therefore, the purpose of this study was to analyze disaster-related deaths in Ishinomaki City during the acute and chronic phases after the disaster to uncover the problems and future issues of indirect deaths in large-scale natural disasters and to develop disaster preparedness in the future.

## 2. Materials and Methods

### 2.1. Design

A retrospective observational study was conducted using public data on disaster-related deaths in Ishinomaki City, Miyagi Prefecture, from March 2011 to January 2021, available from Ishinomaki City Hall.

### 2.2. Details on the Great East Japan Earthquake and Ishinomaki City

**Figure 1:**
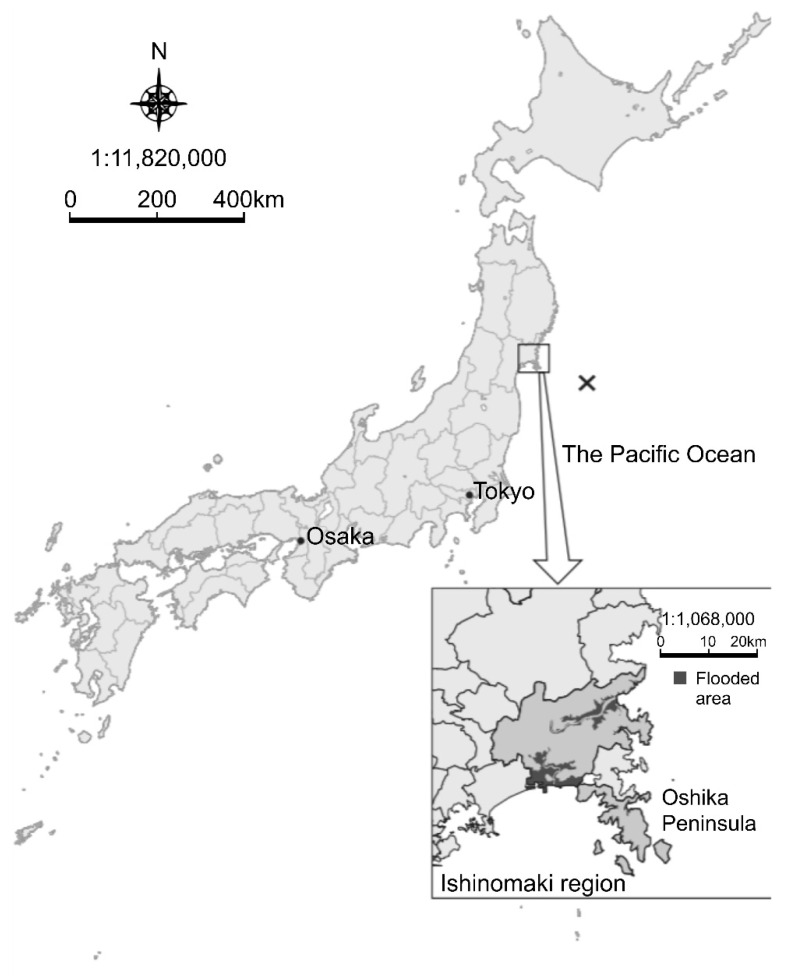
Location of Ishinomaki, Miyagi Prefecture. × indicates the epicenter. The epicenter was approximately 24 km deep off the Pacific Coast of Tohoku, approximately 130 km east–southeast of the Oshika Peninsula in Miyagi Prefecture. The area flooded by the tsunami included 13.2% of Ishinomaki City.

Figure 1 displays the epicenter of the GEJE and the location of Ishinomaki City. The GEJE, which occurred at 14:46 on 11 March 2011, was located about 130 km east–southeast of Oshika Peninsula, Miyagi Prefecture, with an epicenter depth of about 24 km. It was a 9.0 magnitude earthquake [19]. Ishinomaki City is located in the northeastern part of Miyagi Prefecture. Ishinomaki is a port city facing the Pacific Ocean with the second largest population in the prefecture and is also the center of the Ishinomaki metropolitan area. During the GEJE, the maximum seismic intensity observed in Ishinomaki City was 6.0 on the Richter scale [20].

### 2.3. Definition of Disaster-Related Death in Japan

People who succumb to disaster-related death in Japan are defined as follows (Cabinet Office, 2019): “A person who died due to aggravation of injuries caused by a disaster or due to illness caused by physical strain during evacuation, and who was recognized as having died due to a disaster in accordance with the Act on Provision of Disaster Condolence Money (Act No. 82 of 1973). However, it also includes those who were not actually provided with disaster condolence money and excludes those whose address is unknown due to the said disaster)” [15].

GEJE data on disaster-related deaths are based on a collection of cases in which the bereaved families applied to the respective local government, were certified as disaster-related deaths by the certification review board, and for which the families received condolence money. Therefore, even if the death was indirectly caused by a disaster, it was not considered a disaster-related death if there was no application from the bereaved family, or if the submitted documents could not prove that the death was caused by a disaster. However, at present, the only data available on disaster-related deaths in Japan are based on this disaster condolence payment system [16,21].

### 2.4. Disaster-Related Death Certification

Doctors, lawyers, and Ishinomaki City officials were involved in disaster-related death certification in Ishinomaki City.

### 2.5. Data Collection

Anonymized public data on certified disaster-related deaths from March 2011 to January 2021 were obtained from the website of Ishinomaki City Hall. The data included death certificates, living conditions before and after the disaster including welfare information, and some medical record information. The data were publically available to anyone through the Ishinomaki City Hall website [22].

### 2.6. Data Analysis

First, based on death certificates, we descriptively analyzed the direct causes of disaster-related deaths by period and age. Second, Cox regression analysis (forced entry method) was used for all disaster-related deaths to examine the effect of variables. The covariates were age, sex, body wetness due to the tsunami, residence at the time of the disaster, daily living activities at the time of the disaster, evacuation, house damage, and causes of the disaster-related death certification.

The proportional hazard confirmed the distribution of the maximum absolute value of the cumulative Schoenfeld remains using 1000 simulations. All analyses were performed using SAS version 9.4 statistic software (SAS Institute, Inc., Cary, NC, USA). If the two-sided *p* value was less than 0.05, the analysis was considered statistically significant. The sample size calculation was not enforced.

### 2.7. Ethics Statement

The disaster-related death records used in this study were open data anonymized so that individuals could not be identified, and there was no need for ethical approval or patient consent. Open data are available to anyone through Ishinomaki City Hall in Miyagi Prefecture (https://www.city.ishinomaki.lg.jp/cont/10102000/1060/1060.html, accessed on 10 February 2022).

## 3. Results

### 3.1. Characteristics of Disaster-Related Death in Ishinomaki City

**Figure 2:**
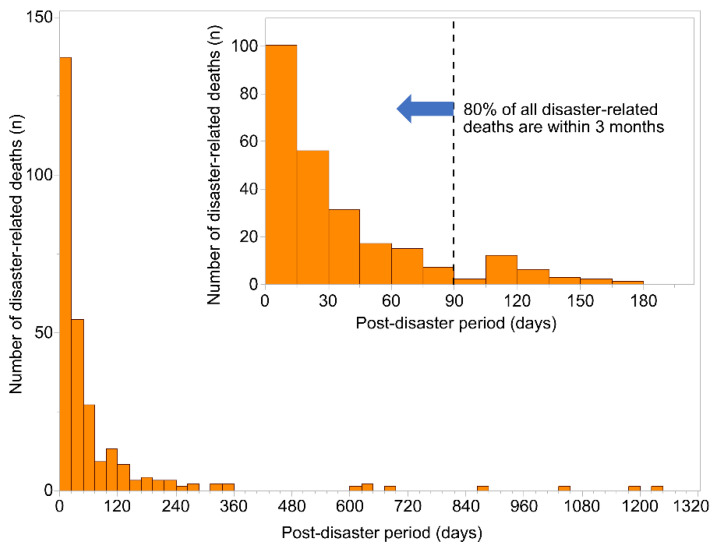
Number of days from earthquake to death.

Table 1 shows the characteristics of disaster-related deaths in Ishinomaki City. The mean age was 79.7 (Standard Deviation [SD] = 13.1) years and included 144 males (52.2%) and 132 females (47.8%). The median time from disaster to death was 24 days (Interquartile Range [IQR] = 9.0–63.8), and about 80% of all disaster-related deaths occurred within three months (Figure 2). Among the direct causes of certified disaster-related deaths, the most common was respiratory disease, with 86 deaths (31.1%). Among respiratory diseases, pneumonia accounted for 74 deaths (86.0% of pneumonia deaths among respiratory diseases). The next most common cause was cardiovascular disease, with 74 deaths (26.8%). Nonmedical factors were the most common factors in the recognition of disaster-related deaths, with 113 deaths (41.0%) attributed to the evacuation environment and 73 deaths (26.4%) to post-disaster mental shock and fatigue.

### 3.2. Direct Causes of Death by Age

**Figure 3:**
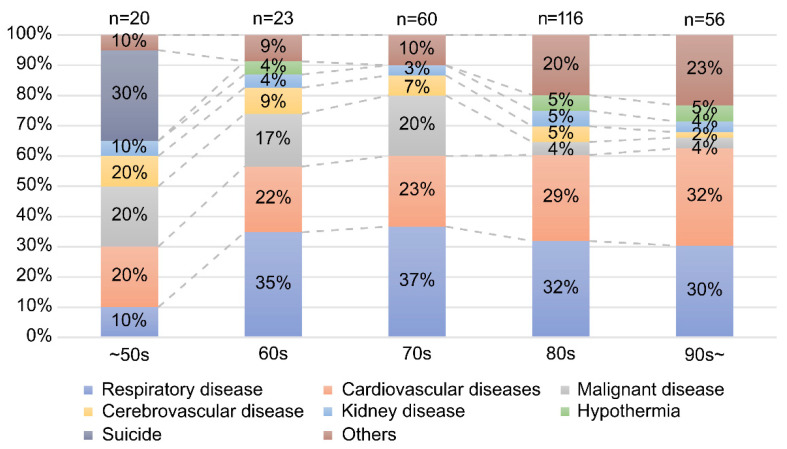
Direct causes of death by age.

Figure 3 shows the ratio of direct causes of death by age. Suicide accounted for the largest ratio (30%) among those under 60 years of age, and respiratory disease accounted for the largest ratio among those who were 60 years of age and older. In the age group of 60 years and above, the second most common cause of death after respiratory diseases was cardiovascular diseases.

### 3.3. Direct Causes of Death by Post-Disaster Period

**Figure 4:**
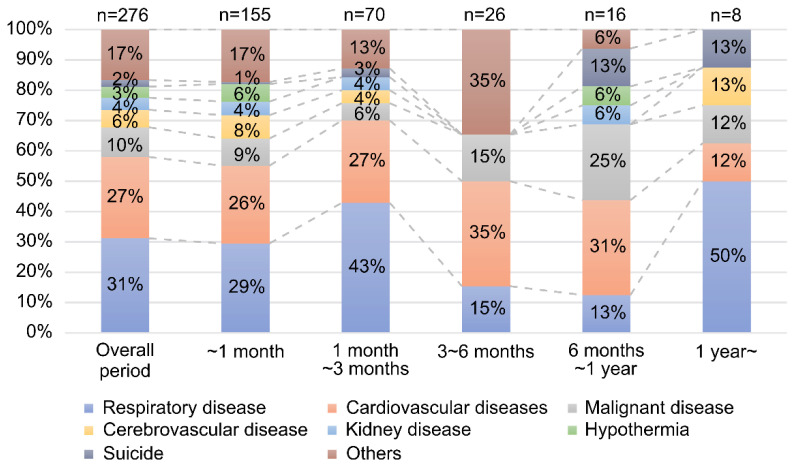
Direct causes of death by post-disaster period.

Figure 4 shows the ratio of direct causes of death by post-disaster period. Respiratory diseases accounted for the largest ratio of direct causes of death in the period of less than three months after the earthquake, which, in turn, accounted for 80% of all disaster-related deaths. On the other hand, the rate of suicide increased six months after the earthquake.

### 3.4. Effect of Variables on Survival Time of All Disaster-Related Deaths

**Figure 5:**
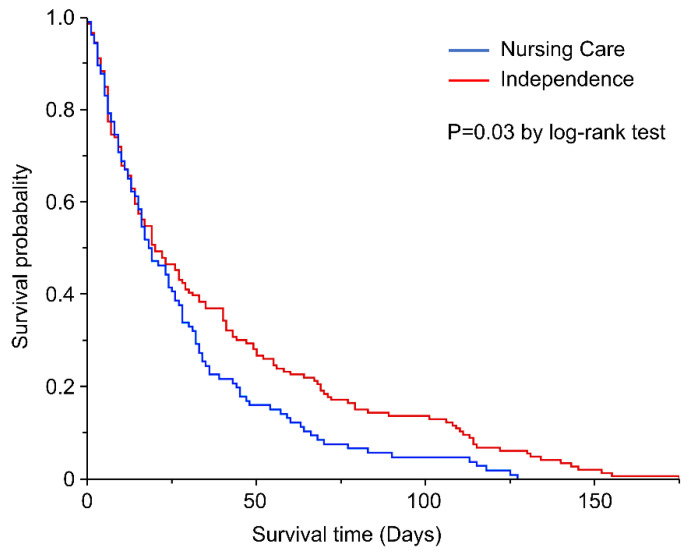
Kaplan-Meier curve of the disaster-related death.

Table 2 and Figure 5 show the effect of variables on the survival time of all disaster-related deaths. The risk of death was statistically significantly higher for those who were certified as being in need of nursing care before the disaster than for those who were independent in activities of daily living (HR, 1.35; 95% CI, 1.03–1.77). Those whose houses were completely destroyed had a significantly lower risk of death from disaster-related causes compared to those whose houses were not damaged (HR, 0.66; 95% CI, 0.46–0.95).

## 4. Discussion

This study was the first to use data on disaster-related deaths in Ishinomaki City, Miyagi, Japan. This study revealed that the direct cause of disaster-related deaths was mainly respiratory diseases such as pneumonia, especially in the elderly, occurring less than three months after the disaster, as shown in Figure 3 and Figure 4. Past studies also reported an increase in respiratory diseases after an earthquake [23]. A cross-sectional study reported an increase in pneumonia cases occurring in coastal areas after the GEJE and an increase in the number of patients hospitalized due to pneumonia [24]. In addition, a significant increase in death due to pneumonia was reported among elderly people (aged 80 years or older) living in nursing homes who needed assistance with daily living [19]. This study showed for the first time that respiratory diseases such as pneumonia were the most common cause of death even in the age group of 60 years and above (below 80 years). In addition, this study is the first report to describe the post-disaster period and to show that deaths due to respiratory diseases were particularly high less than three months after the disaster. The destruction of infrastructure following the tsunami delayed the diagnosis and initiation of treatment and forced people to live in cold environments for long periods of time. Furthermore, the inability to use toilets resulted in diapers being worn by the elderly, who became inactive and may have died due to impaired respiratory function [25]. Thus, in other parts of the world, in disasters involving tsunamis or in cold regions, it is important to prevent respiratory diseases among the elderly immediately after a disaster to reduce post-disaster deaths from respiratory diseases.

Figure 3 and Figure 4 show that suicide was more common among those under 60 years of age, and that the rate of direct cause of death by suicide increased more than six months after the disaster. A study after the Fukushima Daiichi Nuclear Power Plant accident reported that the suicide rate decreased less than one year after evacuation but increased after more than one year [19]. In Miyagi and Iwate prefectures, the most common cause of related deaths, surveyed within one year after the earthquake, was physical and mental burden due to earthquake stress [26]. In Ishinomaki City, Miyagi Prefecture, evacuees began to move into temporary shelters during the six-month post-disaster period. The timing of the increase in the suicide rate in this study coincided with the time of the move to the hypothetical housing, and it is possible that the change in living environment after the evacuation caused the increase in suicide rate. To prevent suicide, it is important to take medium- and long-term measures for each age group. In addition, in large-scale natural disasters, people often change their place of residence after the disaster, and the change in residence associated with the disaster is accompanied by a great deal of stress. Mental health care for suicide prevention is particularly important during the period of environmental change after evacuation.

Table 2 and Figure 5 show that the present study quantified the risk of death of those requiring care in disaster-related deaths. Although the risk of death associated with evacuation in nursing homes was reported during Hurricane Katrina, there are no reports of potential mortality risk for persons in need of care in disaster-related deaths, including in other areas. Dealing with vulnerable groups, such as the elderly and those in need of care, is important in reducing indirect deaths. In the event of a large-scale natural disaster, resources such as people and supplies are limited. Therefore, it is necessary to take measures during normal times, such as establishing welfare shelters that can provide special care to those in need of care and establishing guidelines.

Table 2 also shows that those who escaped direct death but whose houses were completely damaged were at lower risk of disaster-related deaths. After the Hanshin-Awaji Earthquake, stress was higher in the group with greater house damage [27]. In this study, the cause of the relationship between the degree of house damage and the risk of disaster-related deaths is not clear. However, many who avoided direct death but had their houses destroyed lived in shelters. After the GEJE, people lived in their houses covered with sludge and dust from the tsunami. Additionally, elderly people could not evacuate even if they wanted to and were forced to live post-disaster in their houses. These factors may have reversed the degree of damage to houses and the risk of death.

There were several limitations to this study. First, there may be confounding factors that were not measured in this study, including information on internal medication and post-disaster transportation status. Second, the definition of disaster-related deaths in Japan is based on the condolence money system, cases in which the bereaved family applies to the municipality and are recognized are those counted as “disaster-related deaths.” On the other hand, the recognition of disaster-related deaths may be overestimated due to condolences. However, data on indirect deaths due to disasters in Japan can only be obtained based on the condolence payment system. Although analysis of past data is necessary, there is no detailed analysis using the data on disaster-related deaths in Japan. The United Nations Office for Disaster Risk Reduction (UNDRR), in its Sendai Framework for Disaster Risk Reduction 2015–2030, has set a goal of reducing the number of disaster fatalities and has identified understanding, managing, and reducing disaster risks as priority actions to achieve this goal [28]. In addition, the Organization for Economic Co-operation and Development (OECD) shows economic support measures for disasters in each country, suggesting that quantitative analysis is necessary when considering the risk of disaster impact on health [29]. Third, there are limitations in generalizing the results of this study. In the GEJE, the characteristics of damage differed depending on the tsunami and nuclear accidents. In addition, systematic support was provided in Ishinomaki City, whereas the results may have been different in areas where support and acceptance were delayed owing to uneven distribution of support. Disasters are highly regional in nature, and it is important to analyze each region individually. In the future, analysis of data on disaster-related deaths in other affected municipalities may provide evidence to help reduce disaster-related deaths.

## 5. Conclusions

This study is the first report of an analysis of disaster-related deaths in Ishinomaki City, Miyagi, Japan which was damaged by the multiple disasters of the earthquake and tsunami caused by the GEJE. The results showed that the most common direct cause of disaster-related deaths was respiratory disease (e.g., pneumonia), especially if the disease occurred less than three months after the disaster and in people aged 60 years or older. In addition, the rate of suicide was higher among young people and increased six months or more after the disaster. Furthermore, the risk of death in disaster-related deaths was statistically higher among the elderly and those requiring nursing care. Based on these results, in order to reduce disaster-related deaths, it is important to establish evacuation environments and medical care delivery systems that provide special care and treatment for vulnerable disaster victims, such as the elderly and those requiring nursing care, during normal times, and to provide medium- to long-term mental health support for various environmental changes after the disaster. In other words, to reduce indirect deaths, there is a need for continuous proactive medical support and primary health care interventions in the early stages of a disaster. Responding to vulnerable populations during disasters is also important for disaster preparedness in other parts of the world, but further analysis beyond Japan is needed to verify the international validity of the approach this paper proposes.

## Figures and Tables

**Table 1:** Characteristics of all 276 certified disaster-related deaths in Ishinomaki City.

Variables	Overall (*n* = 276)
Age, years (SD)	79.7 (13.1)
Sex, *n* (%)	
Male	144 (52.2%)
Female	132 (47.8%)
Number of days between earthquake and death	
days (IQR)	24 (9.0,63.8)
Direct cause of death, *n* (%)	
Respiratory disease	86 (31.1%)
Cardiovascular diseases	74 (26.8%)
Malignant disease	27 (9.8%)
Cerebrovascular disease	16 (5.8%)
Senility	14 (5.1%)
Kidney disease	11 (4.0%)
Hypothermia	10 (3.6%)
Sepsis	8 (2.9%)
Suicide	6 (2.2%)
Multiple organ failure	5 (1.8%)
Suffocation	4 (1.4%)
Digestive disorders	4 (1.4%)
Trauma	2 (0.7%)
Blood disorders	1 (0.4%)
Others	9 (3.3%)
Body wet by tsunami, *n* (%)	
Yes	75 (27.2%)
No	201 (72.8%)
Residence at the time of disaster, *n* (%)	
Home	209 (75.7%)
Nursing home	38 (13.8%)
Hospital	29 (10.5%)
Activities of daily living, *n* (%)	
Independence	127 (46.0%)
Nursing care	149 (54.0%)
Past History, *n* (%)	
Yes	232 (84.1%)
Yes	44 (15.9%)
Refuge, *n* (%)	
Yes	232 (84.1%)
No	43 (15.6%)
House damage, *n* (%)	
No	63 (22.8%)
Partial	118 (42.8%)
Complete	95 (34.4%)
Reason for earthquake-related death recognition, *n* (%)	
Medical factors	70 (25.4%)
Nonmedical factors (evacuation environment, etc.)	206 (74.6%)

Data display *n*, %; mean (sd); median (IQR).

**Table 2:** Cox regression analysis of disaster-related death.

Variables	Univariable Model			Multivariable Adjusted Model (After Forced Entry)		
	HR	95% CI		HR	95% CI	
Age (per 1 year up)	1.01	1.00	1.02	1.01	1.00	1.02
Female (vs. male)	0.87	0.69	1.11	0.88	0.69	1.13
Places at the time of the earthquake						
Home	reference			reference		
Nursing home	1.08	0.76	1.53	0.96	0.66	1.40
Hospital	1.56	1.05	2.31	1.40	0.89	2.19
Nursing care (vs. independent)	1.38	1.08	1.76	1.35	1.03	1.77
Tsunami damage	1.04	0.80	1.36	1.18	0.88	1.59
Evacuation	0.83	0.65	1.05	1.04	0.79	1.37
House damage						
No damage	reference			reference		
Partial	0.77	0.57	1.05	0.75	0.55	1.04
Complete	0.68	0.49	0.93	0.66	0.46	0.95
Reason for earthquake-related death recognition						
Medical factors	reference			reference		
Nonmedical factors (evacuation environment, etc.)	1.07	0.82	1.41	1.18	0.88	1.59

HR, Hazard ratio; 95% CI, 95% Confidence Interval.

## Data Availability

Not applicable.
